# Growth Arrest Specific 2 (GAS2) is a Critical Mediator of Germ Cell Cyst Breakdown and Folliculogenesis in Mice

**DOI:** 10.1038/srep34956

**Published:** 2016-10-13

**Authors:** J. Philippe York, Yi Athena Ren, Jie Zeng, Bin Zhang, Fang Wang, Rui Chen, Jianqiao Liu, Xuefeng Xia, Pumin Zhang

**Affiliations:** 1 The Third Affiliated Hospital, Guangzhou Medical University, Guangzhou, Guangdong 510150, China; 2 Houston Methodist Research Institute, 6670 Bertner Ave, Houston, TX 77030, USA; 3 Department of Molecular Physiology and Biophysics, Baylor College of Medicine, Houston, TX, 77030, USA; 4 Molecular and Cellular Biology, Baylor College of Medicine, Houston, TX, 77030, USA; 5 Biochemistry and Molecular Biology, Baylor College of Medicine, Baylor College of Medicine, Houston, TX, 77030, USA

## Abstract

In the mouse ovary, the primordial follicle pool is established through a diverse array of signaling pathways and tissue remodeling events. Growth arrest specific gene two (GAS2) is a highly conserved cytoskeleton-associated protein whose *in vivo* function remains unclear. In *Drosophila*, loss of the GAS2 homolog, Pigs, results in infertility. We demonstrate herein that, in the mouse ovary, GAS2 is expressed in the stromal cells surrounding the oocyte cysts on 16.5 dpc, and in stromal cells surrounding growing follicles during juvenile and adult life. We have generated genetically engineered mice with inactivated *Gas2*. *Gas2* homozygous mutant mice are viable but have severely impaired fertility in females, in which oocyte cyst breakdown is disrupted and follicle growth is impaired, with significantly reduced numbers of large antral follicles and corpora lutea. In these mutant mice, the organization of the basal lamina surrounding developing follicles is severely defective at multiple stages of folliculogenesis. We also found that Notch signaling activity was altered in ovaries from *Gas2* null mice around the time of birth and during follicular development later in life. These results indicate that GAS2 is a critical and novel regulator of tissue remodeling in the ovary during oocyte cyst breakdown and folliculogenesis.

Female fertility in mammals is determined by the size of a resting primordial follicle pool, and the highly organized recruitment, growth and death of activated follicles. In mice, the primordial follicle pool is established shortly after birth through a process beginning as early as embryonic day 17.5 that involves fragmentation of germ cell cysts, massive oocyte death, and the reorganization of the remaining oocytes and somatic cells into primordial follicles^1^,^2^. The mechanisms regulating cyst breakdown and the development of a functional and healthy follicle pool are dependent upon interaction between germ cells and surrounding somatic cells, including precursor cells for granulosa cells, theca cells and other stromal cells^2^,^3^,^4^. A diversity of proteins with an array of functions has been implicated in primordial follicle formation. One of the key factors are molecules coordinating cytoskeletal rearrangements and thereby regulating a number of physiological processes, such as the migration of granulosa and theca cell precursors, and meiotic progression and oocyte maturation^1^,^5^,^6^. Organized restructuring of the extra-cellular matrix (ECM) is also important, both during oocyte cyst breakdown and for the integrity and health of growing follicles^7^,^8^. In growing follicles, continuous remodeling of the ECM allows the follicle to expand without breaching the integrity of the basal lamina^9^,^10^,^11^,^12^,^13^. Last but not least, a number of signaling pathways have been implicated in follicle assembly, including steroid hormones, members of the TGF-β super family (such as activin), and the Notch signaling pathway. However, the process of follicle assembly is still not fully understood, and novel regulators await discovery^3^,^14^,^15^,^16^,^17^,^18^.

Growth Arrest Specific 2 (GAS2) is a cytoplasmic protein that interacts with both microtubules and microfilaments through its GAS2 and Calponin homology domains, respectively^19^. The GAS2 protein family contains GAS2 and GAS2-like proteins *Gas2l1*, *Gas2l2*, and *Gas2l3*. GAS2 is proposed to play a role in the dynamic rearrangement of the cytoskeleton during cell proliferation, differentiation and apoptosis^19^,^20^,^21^. GAS2 was initially discovered in mouse fibroblasts, where increase in its expression was associated with serum starvation mediated cell cycle arrest^22^. The protein is a target of Caspase cleavage in its C-terminal domain, and over-expression of its cleaved form induces apoptotic rearrangements of the actin cytoskeleton^19^,^23^. Importantly, GAS2 is widely expressed in developing mouse embryos, particularly during the process of tissue remodeling, and is found in the genitourinary tract (GU)^20^.

In *Drosophila*, loss of function of the fly ortholog for GAS2-like proteins, Pigs, results in fusion of egg chambers and causes infertility^24^. Egg chambers in the *Drosophila* ovary contain germ cells that are surrounded by a monolayer of somatic follicle cells, a functional unit similar to those of a follicle in the mammalian ovary^25^,^26^. There are three types of follicle cells in the fly, polar cells, stalk cells and epithelial cells. Their migration, differentiation and proliferation is critical for the formation of egg chambers and the generation of healthy follicles^25^. Although these follicle cells are not exact structural and functional parallels to the cells that compose mammalian follicles, egg chamber formation in the fly has provided an invaluable tool and model to understand key components of mammalian ovary organogenesis^26^. These key components include, but are not limited to, cell-cell communications between germ cells and somatic cells, individual and collective cell migration, changes in ECM, cell adhesion and cell shape during follicle growth, and the signaling pathways that regulate these processes^27^. One important regulator of somatic cells in the ovary during follicle assembly and folliculogenesis is the Notch signaling pathway, the disruption of which causes severely impaired follicle organization^14^,^28^,^29^. Interestingly, Pigs has been shown to be a downstream effector of the Notch signaling pathway, and plays a role in the regulatory feedback loop that fine-tunes Notch signaling activity^24^.

Based on the foregoing considerations, we hypothesize that 1) GAS2 may be a critical regulator of follicle assembly and follicular growth in the mouse ovary, and 2) it may do so by interacting with the Notch signaling pathway. Results from these studies provide evidence that GAS2 is a novel regulator of the formation and function of ovarian follicles, and that it is indispensible for female fertility.

## Results

### GAS2 is expressed in stromal cells of the ovary

**Figure 1:**
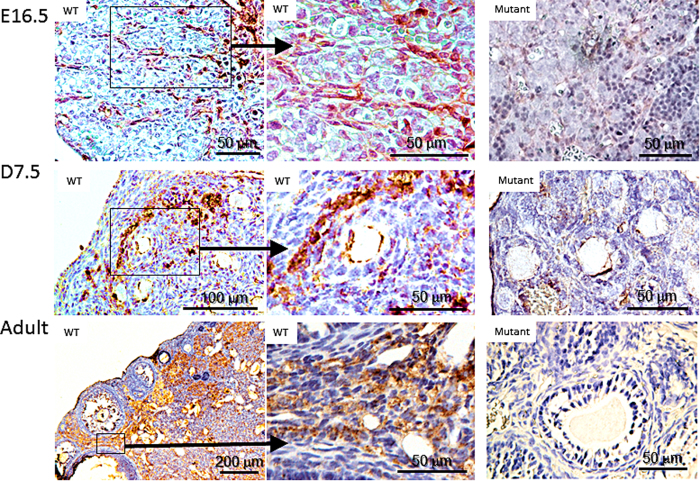
GAS2 is expressed in stromal cells of the mouse ovary. Representative images of staining for GAS2 (brown) on paraffin-embedded ovaries collected at 16.5 dpc (top panels), P7.5 (middle panels), and 6 weeks of age (bottom panel). At all ages examined, expression of GAS2 was observed in the ovaries from wild type mice, but not in those of *Gas2* null mutant mice. In ovaries from wild type mice, GAS2 was expressed in spindle-shaped somatic cells surrounding oocyte cysts at 16.5 dpc; in streams of stromal cells near the cortex of the ovary at P7.5; and in the ovarian stroma surrounding growing follicles in adult mice.

While GAS2 expression in the GU had been previously reported, little was known about the spatial and temporal expression of GAS2 in the mouse ovary. Immuno-staining with GAS2 antibody demonstrated its expression mostly in the ovarian stroma in wild type mice at all ages examined (Fig. 1). At embryonic day (E) 16.5, streams of somatic cells are stained positive for GAS2 in the ovaries of wild type mice (Fig. 1). Close examination revealed that they appear to be somatic cells outlining oocyte cysts. At post-natal day (P) 7.5, GAS2 is expressed in stromal cells near the cortex of the ovary throughout the cortical stroma, particularly between the boundaries of growing follicles and the primordial follicle pool (Fig. 1). In the adult ovary, GAS2 is expressed throughout the stromal tissue surrounding growing follicles.

### Generation of mice with Gas2 inactivation

**Figure 2:**
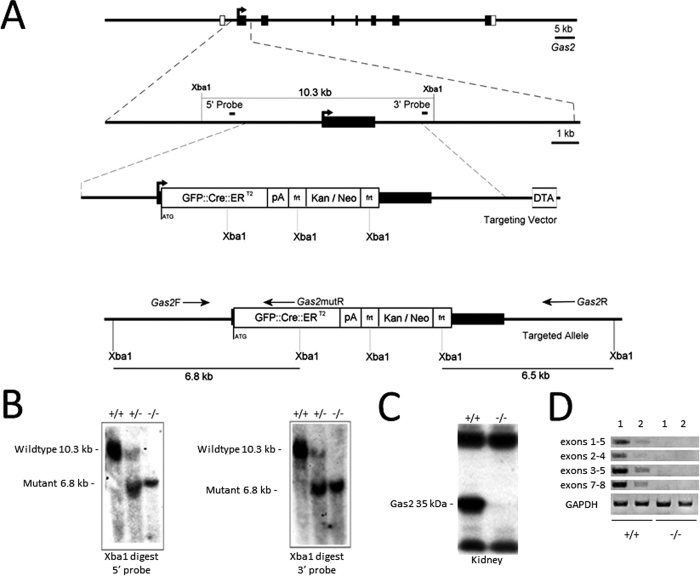
Generation of mice with *Gas2* inactivation. (**A**) Targeted disruption of *Gas2*. The illustration includes *Gas2* exon organization (empty boxes are untranslated regions (UTRs), black boxes are open reading frames), Xba1 restriction sites and the 5′ and 3′ probes used for confirmation of the mutant by southern blot, the targeting vector containing a GFP::Cre::ER^T2^ cassette, poly-A region, and FRT flanked kan/neo resistance, as well as the position of *Gas2*F/R and mutR genotyping primers. (**B**) Southern blot analysis of *Gas2* mice with 5′ and 3′ probes showing wild type bands at 10.3 kb, 5′ mutant band at 6.8 kb, and 3′ mutant band at 6.5 kb. (**C**) Western blot analysis of total kidney protein showing absence of the 35 kDa *Gas2* protein in the mutant mice. (**D**) RT-PCR analysis of mRNA expression in *Gas2* −/− mice. Total RNA was extracted from kidney (1) and ovary (2) of wild-type and mutant mice. Primers amplify between exons 1–5, exons 2–4, exons 3–5, and exons 7–8 of the *Gas2* cDNA. Primers against GAPDH were used as the control.

To determine the developmental and physiological function of GAS2, we generated a *Gas2* null mouse line in which the *Gas2* gene was inactivated by inserting a *GFP:-CreERT2* cassette into the first coding exon of *Gas2* (Fig. 2A). The *GFP-CreERT2* cassette was designed to allow visualization of tissues expressing targeted genes by placing GFP expression under the control of the *Gas2* promoter, as well as, optionally, allowing tamoxifen induced expression of cre recombinase. The targeting vector was introduced into mouse embryonic stem cells and recombinant clones were identified through southern blot analysis. One of the clones was used to generate chimeric mice that successfully transmitted the targeted allele (Fig. 2B). Neither Gas2 messenger RNA (mRNA), nor Gas2 protein could be detected in the *Gas2* null mice by RT-PCR (Fig. 2D) or Western blot (Fig. 2C). *Gas2* null female mice were viable and healthy, but exhibited impaired fertility as discussed below.

### Gas2 deficiency results in subfertility in female mice

**Figure 3:**
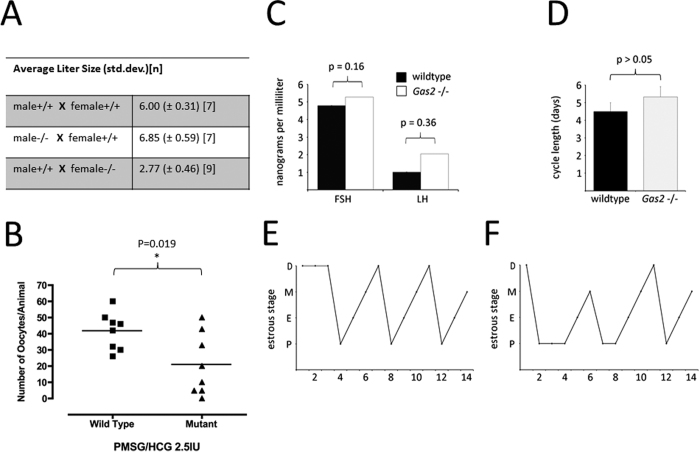
Female *Gas2* null mice are subfertile. (**A**) The number of pups per litter were recorded for six months for breeding pairs of *Gas2* mutant males and females. *Gas2* null males had normal fertility, but females were subfertile. (**B**) Upon superovulation stimulation at six weeks of age, *Gas2* null females released significantly fewer oocytes compared to wild type mice. (**C**) Wild type and mutant proestrus serum levels of follicle stimulating and luteinizing hormones are comparable (FSH *p > 0.05, LH *p > 0.05). (**D**) The length of the estrus cycle in both control and mutant mice was within the normal range (n = 3), as determined by vaginal smear and tracked for fourteen days in the wild type (**E**) and mutant mice (**F**) (D, diestrus; M, metestrus; P, proestrus; E, estrus).

Female mice null for *Gas2* exhibited reduced fertility. The numbers of pups born were recorded for six months (starting from six weeks of age) for breeding pairs of wild type, heterozygote *Gas2* mutant mice and homozygous *Gas2* null mice. Male breeders null for *Gas2* had comparable numbers of pups per litter as wild type breeders (Fig. 3A), but female mice null for *Gas2* had significantly reduced numbers of pups per litter, with an average litter size of 2.9 pups compared to 6 for wild type breeders (Fig. 3A). When oocytes were collected and counted from the ampullae of superovulated mice, female *Gas2* null mice had a significantly reduced rate of ovulation when compared to wild type mice (Fig. 3B). We measured the levels of follicle stimulating hormone (FSH) and luteinizing hormone (LH) in serum samples, but found no apparent difference between wild type and mutant females (Fig. 3C). Consistent with this observation, despite a slightly lengthened estrous cycle and a prolonged proestrous phase in *Gas2* null females (Fig. 3E,F), the difference in estrous cycle length was not statistically different (Fig. 3D)^30^.

### Loss of GAS2 disrupts follicular development

**Figure 4:**
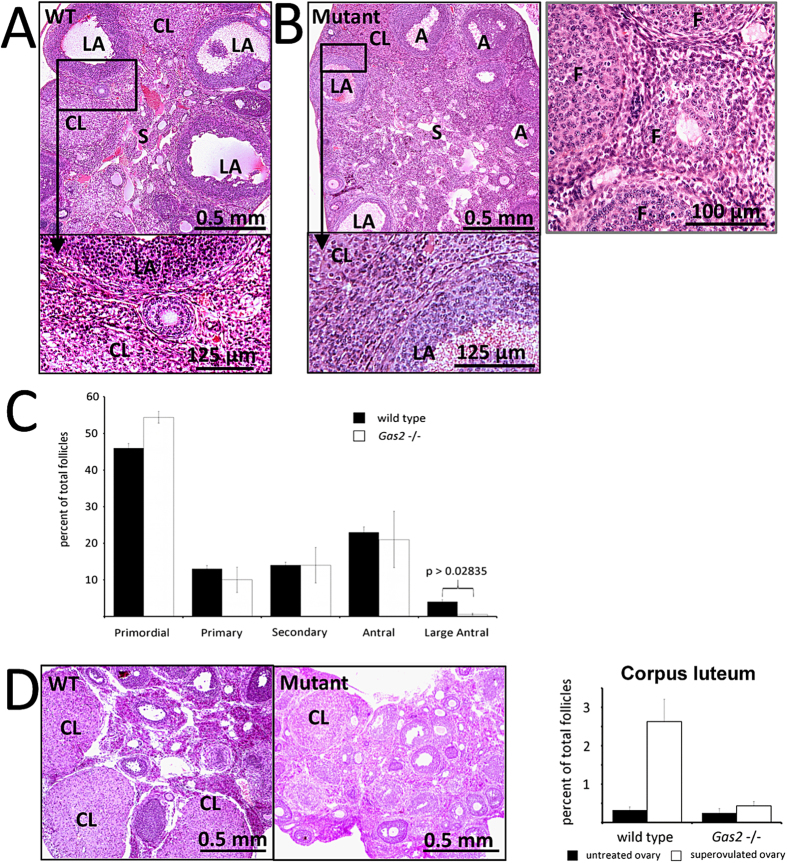
*Gas2* deficiency disrupts follicular development. Representative images of H&E stained sections of ovaries collected from wild type (**A**) and mutant (**B**) mice at six weeks of age. A – Antral Follicle, LA – Large Antral Follicle, CL – Corpus Luteum, S – Stromal tissue. *Gas2* null mice had fewer large antral follicles and corpora lutea compared to wild type. Further examination revealed that compared to wild type mice, follicles and CLs in *Gas2* null females had less defined boundaries and more disorganized stromal cell structures immediately surrounding follicles and CLs. (**C**) The number of follicles at different stages of development was quantified as a percent of the total number of follicles. *Gas2* null females had significantly percentage of large antral follicles (*p = 0.02835 n = 8) (Primordial p = 0.130, Primary p = 0.193, Secondary p = 0.485, Antral p = 0.341, n = 8). (**D**) Ovaries were also collected from wild type and *Gas2* null mice superovulated at six weeks of age and the numbers of CLs were counted. The number of CLs were significantly reduced in the *Gas2* null mutant compared to wild type mice (p = 0.001, n = 10).

Ovaries from six-week-old *Gas2* null mice were similar in size and appearance when compared with those from wild type mice. However, when histological sections of the ovaries were carefully examined, the follicles and corpus luteum in *Gas2* null females had poorly defined boundaries separating them from the surrounding stromal tissue (Fig. 4A,B). These poorly defined boundaries of follicles and corpus luteum in the ovaries of *Gas2* null females are likely a result of disorganized ovarian stromal cells and ECM. To investigate the cause of subfertility in these mutant females, we counted the number of follicles at each developmental stage and calculated their percentage among all follicles counted. When compared with wild type females, the percentages of all other types of follicles are similar, but the percentage of large antral follicles were significantly decreased in *Gas2* null females (Fig. 4C). Therefore, primordial follicle recruitment and the early stages of follicular development are likely normal in the *Gas2* null females, but the formation of the follicular antrum and subsequent development are severely impaired in these mice. When given superovulatory stimulation, very few follicles grew to form antrums, and very few corpora lutea were observed in *Gas2* null females, suggesting that the defects in *Gas2* null mice are ovary-intrinsic (Fig. 4D).

### Loss of GAS2 disrupts the breakdown of oocyte cysts

**Figure 5:**
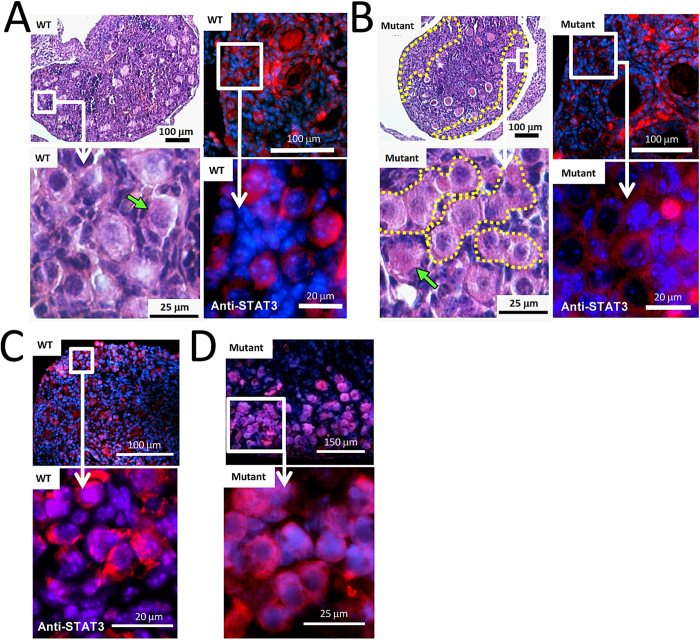
Oocyte cyst breakdown is disrupted in the *Gas2* null mutant mice. At P7.5, the processes of oocyte cyst breakdown and primordial follicle assembly are completed in wild type mice, and the ovarian cortex contains mostly oocytes surrounded by pre-granulosa cells and other somatic cells instead of clusters of oocytes (**A**) (H&E staining). In comparison, large regions of compacted oocyte clusters persist in ovaries of *Gas2* null mice (**B**) (H&E staining), indicating disrupted breakdown of oocyte cysts. Dotted lines in the H&E images outline regions of compacted oocyte clusters. The difference in oocyte cyst breakdown was further illustrated by immunofluorescent labeling of oocytes with anti-STAT3 antibody (red) and nuclei counter staining by DAPI (blue). Oocytes in wild type mice are mostly separated from each other by somatic cells (**A**), but many of them remained immediately next to each other in *Gas2* null mice (**B**). This disrupted breakdown of oocyte cysts in *Gas2* null mice was apparent as early as P1.5 as shown by STAT3 staining in comparison to wild type mice (**C**,**D**).

Based on the role of the GAS2-like protein ortholog Pigs in the fly, we predicted a role for GAS2 in oocyte cyst breakdown and follicular assembly. Oocyte cyst breakdown occurs in mice around the time of birth and completes around postnatal day 4 and 5. By postnatal day 7.5, most oocytes in wild type mice are separated from each other by somatic cells and have been incorporated into individual primordial follicles (Fig. 5A). In contrast, large clusters of oocytes persist in oocyte cysts within the ovarian cortical region in *Gas2* mutant ovaries of the same age (Fig. 5B). Immunofluorescent staining with STAT3, a marker for oocytes in neonatal ovaries, further demonstrated that, at postnatal day 7.5, most oocytes in wild type mice are not in direct contact with each other (Fig. 5C), while clusters of oocytes remain in oocyte cysts in ovaries from *Gas2* null mice (Fig. 5D). We also observed the presence of multiple-oocyte follicles in the ovaries of *Gas2* null mice, with an increased incidence compared to those of the wild type controls, but we did not quantify this observation. Taken together, these data indicate that the loss of *Gas2* disrupts the breakdown of oocyte cysts and the formation of primordial follicles in mouse ovaries.

### Loss of GAS2 expression disrupts organization of the basal lamina during follicle growth

**Figure 6:**
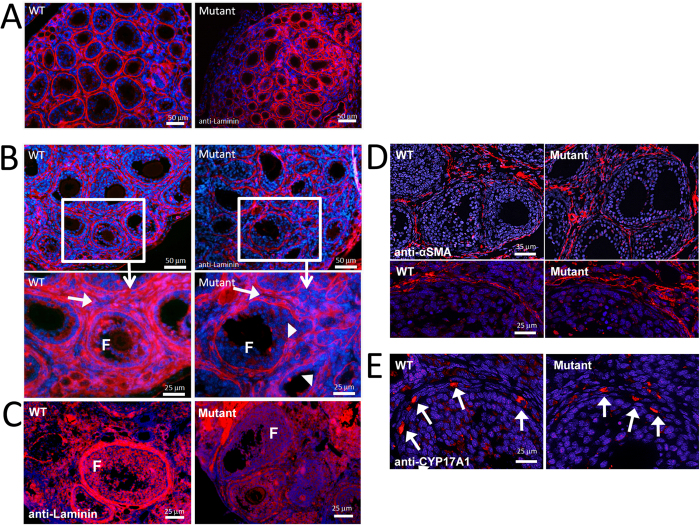
Loss of GAS2 expression disrupts organization of the basal lamina during follicular development. Anti-laminin (red) immunofluorescence labeling of the basement membrane with cell nuclei counterstaining by DAPI (blue) highlights the ECM structure surrounding follicles in the P7.5 wild type and mutant mice (**A**). The basal lamina of the *Gas2* null mutant mice was disorganized compared to wild type mice. At P12.5 this disorganization appeared more severe (**B**). Higher magnification highlights the stromal compartment between growing follicles (arrows), which has less dense and organized laminin staining. Arrowheads point to impaired integrity of the basal lamina in *Gas2* null ovaries compared to wild type. Laminin staining of adult ovaries (**C**) showed that the basal lamina surrounding growing follicles remains poorly organized into adulthood in *Gas2* null mutant females; however this does not appear to be due to a reduction in laminin protein expression (**D**), as determined by Western blot analyses (**D**), 1 = ovary, 2 = kidney). Staining of α-SMA (**E**) and CYP17A1 (**F**) demonstrated that vascular structures and thecal cell differentiation appear normal in the Gas2 null mutant mice.

To characterize the biological processes disrupted in the ovaries of *Gas2* null females, especially in the ovarian stromal tissue and ECM, anti-laminin immunofluorescence was used to examine the organization of the basal lamina in ovaries of P7.5, P12.5, and 6-week old mice (Fig. 6). At P7.5, the basal lamina surrounding follicles of the *Gas2* null females appeared irregular when compared with the wild type, and there appeared to be fewer secondary follicles in these mutant mice (Fig. 6A). At P12.5, the difference in the organization and integrity of the basal lamina was apparent between wild type and *Gas2* null females, with severely disorganized basal lamina and granulosa cell layers observed in the mutant females (Fig. 6B). The observed disorganization of the basal lamina continued into adulthood in the *Gas2* mutant ovaries (Fig. 6C). We also examined other cell types in ovarian stromal tissue, including theca cells and vasculature-associated cells. Staining of α-smooth muscle actin (α-SMA), a marker for vascular smooth muscles, and CYP17A1, a marker for steroidogenic theca cells, showed no apparent difference in expression patterns between wild type and mutant ovaries at six weeks of age, suggesting that these two cell types are not affected by the deletion of *Gas2* (Fig. 6D,E).

### Loss of GAS2 expression alters Notch Signaling activity

**Figure 7:**
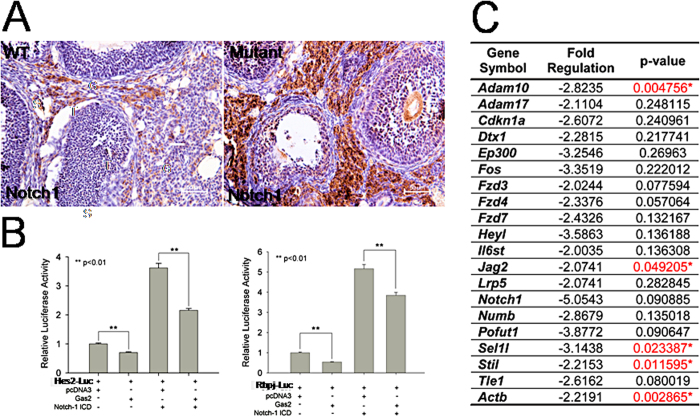
Notch signaling activity is altered in ovaries of *Gas2* null mice. Paraffin embedded wild type and *Gas2* null adult ovary sections were stained for Notch1 (brown). Increased intensity of Notch1 staining was observed in the ovarian stroma of *Gas2* null mutant mice (**A**). The ability of GAS2 to modulate Notch signaling was examined in HeLa cells by luciferase assays using luciferase reporters driven by the promoters of *Hes2* and *Rbpj* (**B**). The first two bars represent endogenous Notch signaling and the second two bars represent Notch signaling induced by a Notch1-ICD construct. Over-expression of GAS2 repressed both endogenous Notch signaling as well as Notch1-ICD induced signaling activity. (**C**) PCR arrays for Notch signaling pathway were performed on neonatal (P1) ovaries from wild type and *Gas2* null mice. Genes with a more than a 2-fold difference in the levels of transcripts are shown. Genes with statistically different levels were highlighted in red (*p < 0.05; n = 4 for each genotype).

Previous studies have shown that loss of Pigs leads to an increase in Notch signaling activity in Drosophila. We investigated whether GAS2 interacts with the Notch signaling pathway during ovarian development. Staining for Notch1 in the adult ovaries of wild type and mutant mice revealed increased expression of Notch1 in the ovarian stroma of *Gas2* null mice (Fig. 7A). Luciferase assays of reporter constructs driven by promoters of *Hes1* and *Rbpj*, both transcriptional targets of Notch signaling, demonstrated the ability of GAS2 to repress their transactivation at both basal and Notch-1 intracellular domain (ICD)-induced states in HeLa cells (Fig. 7B). To obtain a more comprehensive understanding of how GAS2 deletion alters Notch signaling activity *in vivo*, we performed a PCR array designed for the Notch signaling pathway on *Gas2* null and wild type ovaries at postnatal day 1 of age. Strikingly, several major downstream mediators of Notch signaling had significantly reduced levels of mRNA in ovaries from *Gas2* null mice when compared with wild type mice (Fig. 7C). These included *Adam10*, *Jag2*, and *Sel1l*. *Adam10* is a critical regulator of proteolytic activation of Notch signaling activity in multiple developmental process and has been shown lately to control recruitment of pregranulosa cells during folliculogenesis^31^,^32^,^33^. *Jag2* encodes transmembrane receptor of *Notch2*, and *Sel1l* can function as a suppressor of Notch signaling activity in the exocrine pancreas^34^,^35^. Additionally, an important mediator of cell migration and motility^36^, *Actb*, also exhibited a reduced mRNA level in *Gas2* null mice, which may contribute, at least in part, to the disrupted oocyte cyst breakdown in *Gas2* null mice. Together, these results demonstrate interactions between GAS2 and the Notch signaling pathway during multiple stages of ovarian development.

## Discussion

Development of the mammalian ovary is a continuous but multi-staged process that involves complex interactions between multiple cell types and molecular pathways. Using a genetically engineered mouse model, we investigated the *in vivo* functions of a novel regulator during follicle development, GAS2. GAS2 is highly conserved across species and its ortholog Pigs regulates egg chamber formation in *Drosophila* ovaries. We have shown that GAS2 is mainly expressed in the somatic compartment of the ovary, either surrounding the perinatal oocyte cyst or surrounding postnatal growing follicles. Female *Gas2* null mice are subfertile and exhibit disrupted oocyte cyst breakdown, a reduced number of large antral follicles and corpora lutea, and severely impaired organization of the basal lamina surrounding growing follicles. Furthermore, we demonstrate dysregulated Notch signaling activity in *Gas2* null neonatal and adult ovaries, highlighting interaction between GAS2 and the Notch signaling pathway during ovarian development. Collectively, our findings provide evidence of an indispensible role of GAS2 and ovarian somatic cells in the breakdown of oocyte cysts around the time of birth and in follicular ECM remodeling later in life.

The consistent expression of GAS2 in the ovarian stromal cells throughout the development of the ovary suggests its functional importance. Indeed, *Gas2* null females are subfertile. In the adult ovary from *Gas2* null mice, follicles were present at all stages of development; however, a number of abnormalities were observed: 1) the numbers of large antral follicles and corpora lutea were reduced, 2) the stromal supporting structure of follicles was disorganized, with disrupted ECM and boundaries separating follicles and CLs from one another, and 3) superovulation of the *Gas2* null mutant females did not rescue these phenotypes, withreduced total number of ovulated oocytes compared to wild type mice. The fact that serum levels of FSH and LH, and estrous cyclicity were not significantly altered in the *Gas2* null mutant females also suggests that reduced fertility in these mice may be attributed to intrinsic ovarian defects related to follicular growth and ovulation in these mice. Loss of the GAS2 ortholog Pigs resulted in infertility in *Drosophila*^24^. In the PIGS mutants the egg chambers exhibited a number of defects, including an increase in the number of observable germ cells, egg chamber fusions, and aberrant cell morphology^24^. Other than *Gas2* itself, the GAS2 family also contains GAS2-like proteins *Gas2l1*, *Gas2l2* and *Gas2l3*^37^. Microarray datasets from previous studies provided evidence that *Gas2l1* and *Gas2l3* are expressed in the mouse ovary at comparable or higher levels to that of *Gas2* both at the time around birth and in preovulatory period^38^,^39^. Together with GAS2, these proteins all share a GAS2-related domain and a putative actin-binding calponin homology domain^40^. In contrast to the mouse, PIGS is the only Gas2-like protein in the fly. It is possible that functional redundancy provided by the other GAS2-like family proteins in the mouse explains the relatively mild phenotype observed in the *Gas2* mutant mice when compared to the loss of PIGS in *Drosophila*.

Our studies provide novel evidence supporting the role of follicle basal lamina as an indispensible regulator of follicular development and ovulation. Continued remodeling of the ovarian basal lamina is critical for follicle growth, ovulation, and corpora lutea formation^41^. The follicular basal lamina has been proposed to regulate trafficking of growth factors and plasma proteins, thus modulating the formation of a large follicle antrum^7^. Previous *in vivo* studies demonstrate that dysregulation of factors such as Kit ligand or ADAMTS-1 result in defective basal lamina organization and reduced fertility^9^,^13^,^42^. In *Gas2* null ovaries, follicular basal lamina is impaired from the onset of follicle activation, as indicated by the lack of a clear boundary between follicles, as well as severely abnormal basal lamina organization. Because of the impaired basement lamina, despite the ability to develop to antral stage, the number of large antral follicles and the rate of ovulation is reduced in mutant compared to wild type females. In ovaries of the *Gas2* null mice, follicular basement lamina is disorganized from the early onset of follicle development (Fig. 6C), but the functional consequence of this basement lamina defect is most easily detected at the transition between antral to large antral follicles, and during the process of ovulation (Fig. 5). These observations indicate that, in *Gas2* mutant mice, defects in the basement lamina occur from a very early age, and leading to the adult ovarian phenotype later in life when more dynamic remodeling processes take place.

Because GAS2 is mainly expressed in the stromal cells of the ovary, it is most likely that the abnormal basal lamina in these mice is due to defects in these cells, thus highlighting the importance of stromal cells in basal lamina formation and organization during follicle development. As in many other tissues and organs, the role of stromal cells during development, tissue homeostasis and tumoriogenesis are not well understood. One reason is that all of the cell types existing in a particular stromal tissue have not been thoroughly defined, and the function of each cell type at a specific development stage of the tissue is often unclear. Here, we have shown that theca cells and vascular cells are unlikely to be responsible for the defective basal lamina in *Gas2* null mice. It would be of future interest to identify the cell types that are critical for follicular basal lamina organization, and *Gas2* null mice may provide a novel and informative tool for this line of studies.

We have demonstrated that GAS2 interacts with the Notch signaling pathway during multiple stages of follicle development. Supporting evidence includes: 1) GAS2 and Notch localization. Before and around the time of birth, GAS2 is expressed in somatic cells surrounding oocyte cysts, coincident with active Notch signaling as indicated by fluorescent reporter expression driven by the Rbpj/CBP1 promoter^29^. In adult ovaries, GAS2 is expressed in somatic cells surrounding growing follicles, and Notch1, Notch4 and Jagged1 are expressed in endothelial cells and vascular smooth muscle cells in the ovarian stroma at both follicular and luteal phases^43^. 2) Transcriptional regulation of Notch signaling activity by GAS2. Luciferase assays using reporters for Notch signaling activity demonstrated suppression of Notch signaling activity by GAS2 in Hela cells, and decreased levels of transcripts for critical mediators of the Notch signaling pathway genes in neonatal ovaries from *Gas2* null mice compared to wild type. The seemingly contradictory effects of GAS2 on expression levels of Notch pathway components is likely due cell type- and developmental stage-specific interactions between GAS2 and the Notch signaling pathway.

Interactions between GAS2 and the Notch signaling pathway may account for at least some of the phenotypes observed in the *Gas2* null females. Disruption of the Notch signaling pathway has been shown to have a negative impact on mouse fertility. Inhibition of Notch signaling in *ex-vivo* organ culture with gamma secretase inhibitors, as well as conditional *Notch2* deletion in the somatic cells of the ovary, leads to a reduction of primary follicle formation and a delay in oocyte cyst breakdown^14^,^28^. One critical mediator of Notch signaling, HES1, is expressed in the somatic cells of embryonic and neonatal ovaries, and its level of expression is tightly associated with the number of both pre-granulosa cells and oocytes^14^,^15^. Utilizing a Notch reporter mouse line and conditional transgenic mice, Vanorny *et al*. elegantly demonstrated that the Notch pathway functions as a critical coordinator between oocytes and ovarian somatic cells, both during primordial follicle assembly and during follicle development^29^. Interestingly, *Adam10*, a gene with significantly reduced transcript expression in the neonatal ovaries of *Gas2* null mice compared to controls (Fig. 7), was recently reported to regulate pregranulosa cell recruitment and follicle assembly^33^. Currently, we cannot exclude mechanisms other than Notch signaling, such as direct interaction with actin filament, that may mediate the effect of GAS2 on ovarian development and future studies will be needed for a better understanding.

In conclusion, we have demonstrated that GAS2 is an important mediator of oocyte cyst breakdown, follicle assembly, and follicular development in mice. GAS2 is expressed in the somatic cells of the mouse ovary at multiple stages of ovarian development. *Gas2* null mutant females are viable, but have severely impaired fertility. In these mutant mice, oocyte cyst breakdown is disrupted; the basal lamina, as well as boundaries between growing follicles and CL, are disorganized; and the number of large antral follicles, as well as the rate of ovulation, are reduced compared to those of wild type mice. We have provided evidence that GAS2 interacts with the Notch signaling pathway during ovarian development, and that alteration in Notch signaling activity may mediate some functions of GAS2 during primordial follicle pool assembly and follicular development. Taken together, these results indicate that GAS2 is a novel and indispensible regulator of female fertility.

## Methods

### Generation of the Gas2 null mice

To disrupt *Gas2*, we designed a targeting vector by the insertion of a *GFP-CreERT2* cassette into the starting codon of the *Gas2* gene. The targeting vector was constructed using the homologous recombination method and introduced into AB2.2 mouse embryonic stem cells. The recombinant clones were screened with Southern blot analysis. One recombinant clone was used for blastocyst injection and the resultant chimeric mice were crossed with C57BL/6 mice to obtain germline transmission of the targeted allele. For geneotyping and selecting mice carrying the mutant allele, we designed the following PCR primers: Gas2F, 5′ accaagtggctgactaatag 3′; Gas2R, 5′ aggacactctcagaggagg 3′; Gas2mutR, 5′ gtccagctcgaccaggatgg 3′. Gas2F/R amplifies a 435 bp product from the wild type allele, and Gas2F/Gas2mutR amplifies a 272 bp product from the mutant allele. All animals were housed under a 14-hour light/10-hour dark schedule in the Center for Comparative Medicine at Baylor College of Medicine and provided food and water *ad libitum*. All animals were maintained according to the National Institutes of Health (NIH) Guide for the Care and Use of Laboratory Animals and approved by the Animal Care and Use Committee at Baylor college of Medicine.

### Fertility analysis

Mating pairs were set up when the mice reached six weeks of age. Pups were counted at birth and the number of births and pups per litter were recorded for each mating pair. To induce superovulation, eight wild type and eight mutant female mice were given pregnant mare serum gonadotropin (PMSG) at four weeks of age followed by human chorionic gonadotropin (hCG) 48 hours after PMSG. Fifteen hours after HCG administration, oocytes were flushed with phophate-buffered saline (PBS) from ampulla and counted. Estrous cycle was determined *via* vaginal smear. 25 μl of PBS was pipetted five to six times at the vaginal orifice, and a drop of the resulting fluid was placed on a microscope slide. The cells suspended in the fluid were observed at 100x without further contrast staining. Determination of cycle stage was performed using cell morphology and density according to standard definitions^30^,^44^. Three wild type and three mutant mice were followed for two weeks (approximately three cycles).

### Hormone measurements

Blood was collected *via* heart puncture from six-week-old mice anaesthetized with isoflurane during estrus (determined as described above) and spun down in a BD Microtainer serum collection tube (BD biosciences, San Jose, CA). All sera were then shipped on dry ice to the University of Virginia Core Laboratory for hormone measuring.

### Histology and immunohistochemistry

Ovaries were fixed in 4% paraformaldehyde (PFA) and embedded in paraffin. 5-μm sections were processed for staining with hematoxylin and eosin (H&E) or for immunofluorescent (IF) or immunohistochemistry (IHC) analyses. Antibodies used were: Anti-GAS2 (ab55076-100) from Abcam (Cambridge, MA, USA); anti-laminin (L9393) from Sigma (St. Louis, MO, USA); Notch1 (AM00349PU-N) from Acris Antibodies, Inc.(San Diego, CA, USA) Notch 2 (SC-5545) from Santa Cruz (Santa Cruz, CA, USA); and Stat3 (#9132) from Cell Signaling Technology (Danvers, MA, USA). Images were acquired on a Nikon E800 using a Spot RT3 camera (Diagnostic Instruments Inc, Sterling Heights, MI, USA). Confocal images were acquired on a Nikon A1 confocal imaging system and further processed and analyzed identically for every sample.

### Luciferase assay

HeLa cells were transfected by Lipofectamine 2000 reagent (Invitrogen, Carlsbad, CA, USA) using 2 μg of the *Hes1* and *Rbpj* luciferase reporter constructs (generous gifts from Dr. Raphael Kopan) with 2 μg of empty vector pcDNA3 or *Gas2* construct according to the recommended conditions by the manufacturer. A truncated Notch-1 construct was used for over-expressing Notch1 intracellular domain (ICD). Cells were harvested 24 hours after transfection and luciferase activity was measured using a dual-luciferase kit according to the manufacturer’s instructions (Promega, Fitchburg, WI, USA). Results present mean ± SD of three independent experiments performed in triplicates.

### Assessment of follicle types and numbers

Follicle staging was determined based on Pedersen’s system as described previously^45^,^46^. Follicles at each major developmental stage were counted in every tenth section and was only counted if an oocyte was visible in the plane of the section. Three wild type and three mutant age matched adult mice were assessed.

### Western blot analysis

Tissue samples were homogenized and lysed in RIPA (Bio-Rad Laboratories, Hercules, CA, USA) buffer, run on a 12% polyacrylamide gel, and wet transferred to Bio-Rad PVDF membrane (Bio-Rad Laboratories) according to the standard protocol and chemiluminescently visualized using HRP-conjugated secondary antibodies from PerkinElmer (Waltham, MA, USA). Anti-GAS2 (ab55076-100) from Abcam (Cambridge, MA, USA) and anti-laminin (L9393) from Sigma-Aldrich (St. Louis, MO, USA).

### RT-PCR analysis

Total RNA was extracted from kidney and ovary tissue by homogenization in TRIzol, followed by extraction of RNA with phenol chloroform PH 4.7. First Strand synthesis was performed with SuperScript^®^ III First-Strand Synthesis System for RT-PCR (Invitrogen, Carlsbad, CA, USA). PCR was carried out using primers (F1 5′-TGGATAATGGTGCCTTGCTCT-3′, R1 5′-GCAGAACCAGGCCTTCAGATT-3′) (F2 5′-CATGAAGCTAATTTGCTGCCGA-3′ R2 5′-TTCTTCAGTGGTAGGGTCTTGG-3′) (F3 5′-GCCAAGACCCTACCACTGAAG-3′, R3 5′-CGACCAAGCTCTAGCAAACA-3′) (F4 5′-GGCTCTCTCAAGGTCGATACC-3′, R4 5′-TCCTTCAGGGTTGGGGATTTG-3′) (mGAPDHF 5′-GCATTGTGGAAGGGCTCA-3′, mGAPDHR 5′-AGGCGGCACGTCAGATC-3′).

### Statistical Analysis

Follicle counts, primordial follicle counts, oocyte counts and litter size were analyzed by unpaired t-tests, and a two-tailed p < 0.05 was considered statistically significant.

## Additional Information

**How to cite this article**: York, J. P. *et al*. Growth Arrest Specific 2 (GAS2) is a Critical Mediator of Germ Cell Cyst Breakdown and Folliculogenesis in Mice. *Sci. Rep.* **6**, 34956; doi: 10.1038/srep34956 (2016).
